# Prevalence and correlates of neurocognitive impairment and psychiatric disorders among schoolchildren in Wakiso District, Uganda: a cross-sectional study

**DOI:** 10.12688/wellcomeopenres.17005.2

**Published:** 2022-09-29

**Authors:** Margaret Nampijja, Wilber Sembajjwe, Harriet Mpairwe, Richard Mpango, Eugene Kinyanda

**Affiliations:** 1 MRC/UVRI and LSHTM Uganda Research Unit, Entebbe, Uganda; 2 Maternal and Child Wellbeing Unit, African Population and Health Research Center, Nairobi, Kenya; 3 London School of Hygiene and Tropical Medicine, Keppel Street, Bloomsbury, London, London, WC1E 7HT, UK., UK; 4 Department of Mental Health, School of Health Sciences, Soroti University, Soroti, Uganda; 5 Department of Psychiatry, College of Health Sciences, Makerere University, Kampala,, Kampala, P.O. Box 7072, Kampala, Uganda

**Keywords:** neurocognitive, psychiatric, disorders, children, adolescents, non-clinical

## Abstract

**Background: **There is limited data on the burden of mental disorders among children in the general population in Africa. We examined the prevalence and correlates of neurocognitive and psychiatric disorders among schoolchildren in Uganda.

**Methods:** This cross-sectional study enrolled 322 schoolchildren aged 5-17years in Wakiso, Uganda. We assessed for neurocognitive impairment using the Kaufmann-Assessment-Battery, and psychiatric disorders (major-depressive-disorder (MDD), attention-deficit-hyperactivity-disorder (ADHD), generalised-anxiety-disorder (GAD), and substance-use-disorder (SUD)) using the parent version of the Child and Adolescent Symptom Inventory-5, and Youth Inventory-4R Self Report. Prevalence and risk factors were determined using respectively descriptive statistics, and univariable and  multivariable logistic regression.

**Results: **Twenty-five participants (8%) had neurocognitive impairment. Nineteen (5.9%) participants had MDD, nine (2.8%) had ADHD, seven (2.2%) had GAD, 14 (8.6%) had SUD; and 30 (9.3%) had any psychiatric disorder. Among the exposure variables examined in this study, including asthma, age, sex, grade of schooling, type of school and maternal and father’s education and family socio-economic status, only asthma was associated with the disorders (MDD).

**Conclusions: **The relatively high burden of mental disorders in this general population of children warrants targeted screening of those at risk, and treatment of those affected. Further, future studies should extensively investigate the factors that underlie the identified psychiatric disorders in this and similar general populations.

## Introduction

Globally, there is a significant burden of mental health problems among children and adolescents with a worldwide prevalence of approximately 20% ^1– 4^. The problem is higher in low-income countries where poverty, diseases, and conflict increase the risk for these disorders, and yet access to mental health services is limited ^1, 5^. In Uganda, for instance, studies have shown a high (about 18%) prevalence of neurocognitive impairment and psychiatric disorders among children and adolescents living with HIV/AIDS and in areas affected by civil war (northern Uganda) ^5– 10^. About 17% of children perinatally infected with HIV are reported to suffer neurological disorders ^11^ and around the same proportion (18%) have psychiatric disorders ^9^. Among adolescents in northern Uganda and particularly those that were abducted during the war, there was a high prevalence of specific psychiatric disorders including post-traumatic stress disorder (26.8%), major depression (19.5%), and generalized anxiety disorder (13.4%) ^5^.

Thus, a lot is known with regards to the burden of neurocognitive and psychiatric problems in children with HIV or exposed to conflict. However, there is limited data on the prevalence and risk factors of these disorders in the non-clinical and non-vulnerable (general) populations. Such populations are assumed to be healthy since they are not exposed to any known overt risk factors for neurocognitive and psychiatric disorders, yet they may carry genetic and perinatal vulnerabilities to these disorders ^12– 16^ or may have been exposed to traumatising events within their families ^17^. Research carried out in the UK that examined risky behaviour among young people revealed that there is a shared risk for mental disorders regardless of whether one is vulnerable or not ^18^. It is possible that effects of disease, conflict and other adverse exposures are superimposed upon this essential vulnerability ^2, 18^.

Research and intervention programs have focused on children at risk of mental health problems because of a major exposure such as a serious medical condition (e.g. HIV), or adverse social circumstances (e.g. war, violence) ^19, 20^. Absence of data on the mental disorders in the general population means that many children may be battling with mental health problems that are not recognised ^18^. The child and adolescent mental health policy in Uganda recommends screening for mental, neurological and substance problems among school-going children and adolescents ^1^, however this has not been implemented implying that any children suffering from these disorders are not identified and therefore do not get treated ^1, 21^. Failure to address neurocognitive and psychiatric challenges among children and adolescents may derail attainment of Sustainable Development Goal (SDG) targets including universal access to quality education (Goal 4) and minimising child abuse and neglect (Goal 16) ^22^. This may consequently hold back the potential of this generation and in turn lock them in a vicious cycle of poverty. Epidemiological data on mental disorders among children in the general population are therefore critical. They will generate reference data for clinical populations, but also for monitoring trends in the burden of the disorders over time. Such data will inform the planning of appropriate interventions to respond to the problem in a timely manner and ultimately promote the mental wellbeing and productivity of affected individuals. The current study examined the prevalence of neurocognitive impairment and psychiatric disorders in a sample of non-clinical schoolchildren and adolescents living in a peri-urban setting in Wakiso, Uganda.

## Methods

### Design and setting

This work was nested within a case-control study that was investigating risk factors for asthma among schoolchildren in Uganda (SONA) ^23^. SONA was conducted among schoolchildren in Entebbe Municipality and Katabi zone in Wakiso District, central Uganda, a predominantly peri-urban area. All schools, primary and secondary, in this pre-determined study area were approached for SONA study between May 2015 to July 2017, and 96% of these participated ^23^. We nested the mental health sub-study in SONA, as a cross-sectional study to estimate the prevalence and risk factors of neurocognitive and psychiatric disorders among schoolchildren in a peri-urban setting.

### Participants

For this mental health study, we recruited schoolchildren enrolled into the SONA study between March and August 2016, from a total of 41 primary and secondary schools. The mental health sub-study was done in 41 schools. The number of students enrolled per school varied between 1 and 35. Ten of the 41 schools were government-supported, while 31 were privately owned.

### Eligibility (inclusion and exclusion criteria)

**Figure 1.**
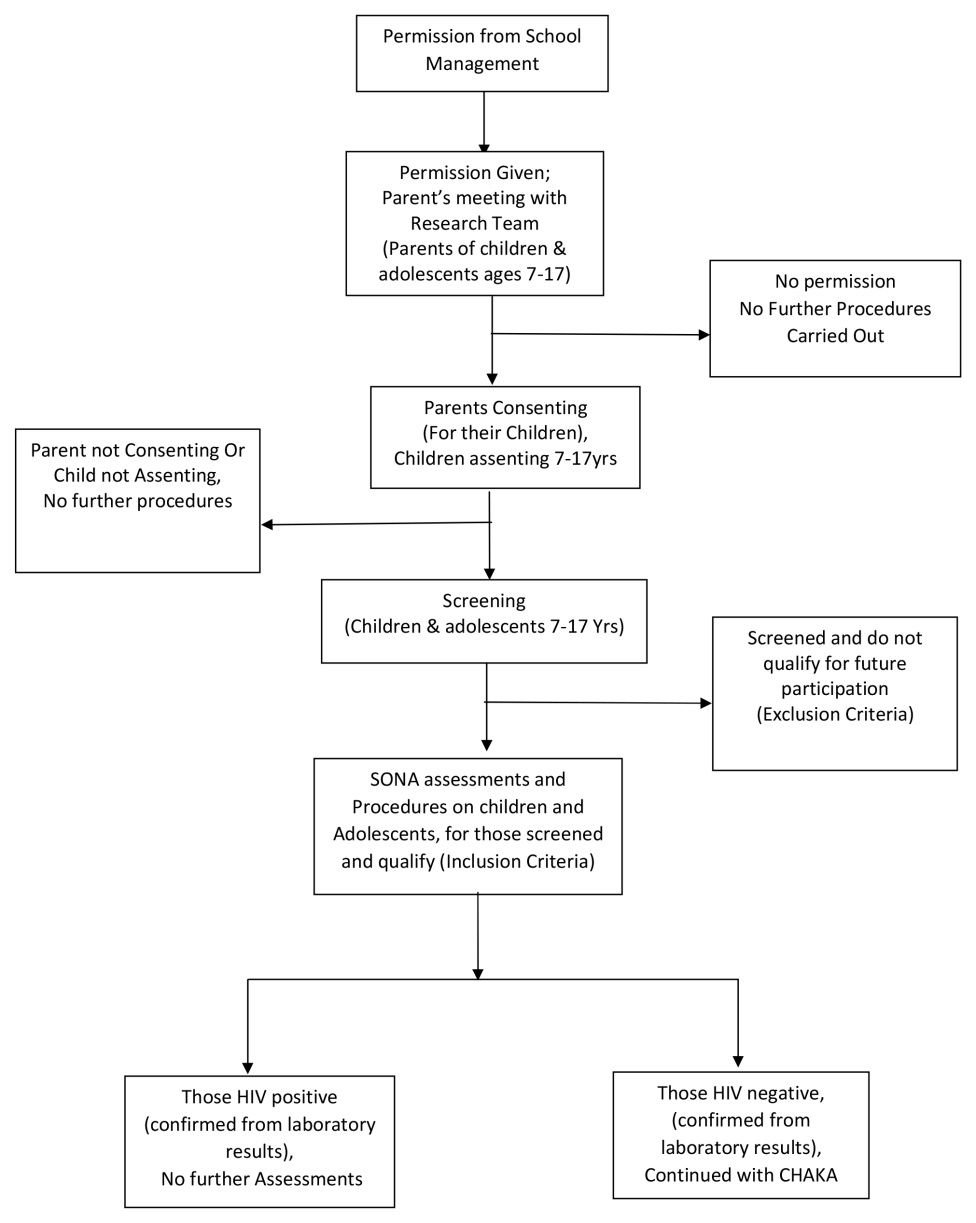
Recruitment flow chart for SONA /CHAKA.

For the SONA study, all children with asthma symptoms were eligible and twice the number of children without asthma were randomly selected from the class register, using the random numbers generator programme in STATA (StataCorp, Texas, USA). A total of 1702 participants were recruited in the SONA study. For the mental health sub-study, all SONA participants enrolled between March and August 2016 and interested in participating in the sub-study were eligible. This included schoolchildren with and without asthma. Children were excluded if the parent/guardian was not available to provide written informed consent and to answer the additional questionnaires for this sub-study. This is represented in the recruitment flow chart diagram ( Figure 1).

***Sociodemographic and health data collection.*** Sociodemographic data including children’s age, sex, schooling information (including the status of the school based on the amount of school fees paid), and mothers’ and fathers’ highest education level were collected using a questionnaire which was administered to the children. Asthma was doctor-diagnosed as per the SONA protocol ^23^.

***Assessing for psychiatric disorders.*** The psychiatric diagnoses were determined using the parent version (5–17 years) of the Child and Adolescent Symptom Inventory-5 (CASI-5) ^24^. This structured diagnostic interview was used to elicit the following DSM V disorders: attention-deficit hyperactivity disorder of the inattentive type (ADHD-I), attention-deficit hyperactivity disorder of the hyperactivity-impulsive type (ADHD-HI), attention-deficit hyperactivity disorder-Combined (ADHD-C), generalised anxiety disorder (GAD), major depressive disorder (MDD), and substance use disorder (SUD). The CASI-5 also provides a global psychological assessment score for the children. The Youth Inventory-4R (YI-4R)- Self Report ^25^ was also used. The criteria for assessing the disorders looked at both CASI-5 and/or YI-4R. The whole spectrum of psychiatric disorders assessed by both tools were examined, however for the analysis we focused on four psychiatric disorders i.e. ADHD (all forms), GAD, MDD, and SUD (tobacco, marijuana, or illegal drugs) as only these were present in the study population; the rest were absent.

The CASI-5 (Parent version) was administered to parents/guardians of children (5–11 years of age) at the schools of their children over the weekends. The YI-4 R was self-administered to youths (12–18 years). Younger children did not complete this measure.

The disorders considered under the CASI-5 and YI-4-R were MDD, ADHD, GAD, separation anxiety disorders, social phobia, eating disorders (Anorexia and Bulimia Nervosa), Post Traumatic Stress Disorder (PTSD). Bipolar affective disorders, conduct disorders, oppositional defiant disorders, psychosis, Tics, somatic symptom disorder (SSD) and substance use disorder(SUD) (one item on the CASI-5, category O was used to screen for SUDs). Additionally, the CASI-5 also screened for autism spectrum disorder (ASD), Enuresis, Encopresis and excoriation disorder. The tool was culturally adapted and translated to Luganda the predominant language in the study setting.

Assessments were conducted at school by two psychiatric clinical officers (PCOs) who had training and experience in administering the different tools. Assessments were conducted for about 45 minutes. Children/adolescents identified to have emotional and behavioural disorders were given initial attention by the PCO but those with persistent symptoms were referred to Entebbe Hospital or Butabika Hospital for further management.

***Assessing neurocognitive functioning.*** Neurocognitive functioning was assessed using the Kaufmann Assessment Battery (KABC-II) which has previously been validated in Uganda by Bangirana and colleagues ^26^. The KABC-II was used to measure performance of participants on Sequential Processing, Simultaneous Processing and Planning domains of intellectual ability. Assessments were conducted at school by two PCOs who had training and experience in administering these tools. These were supervised by a senior clinical psychologist. Individual assessment lasted about 40 minutes. Data collection was done using pre-coded questionnaires which were double entered into OpenClinica open source software version 3.1.4 (OpenClinica LLC and collaborators, Waltham, MA, USA).

### Ethical approval and consent to participate

This study was approved by the Uganda Virus Research Institute Research and Ethics Committee (reference number GC/127/14109/481), and the Uganda National Council for Science and Technology (reference number HS 1707). The ethical approvals and consent were obtained for the overall SONA study, which contained information about this sub-study. All participants’ parents or guardians provided written informed consent (or witnessed thumb print). In addition, children aged eight years and above provided written informed assent to participate in the study. In addition, we obtained permission from the head teachers and education officials from Wakiso district and Entebbe Municipality to conduct the study within the schools.

### Statistical considerations

***Sample size calculation.*** The mental health sub-study was observational and exploratory to measure the prevalence of neurocognitive impairment and psychiatric disorders among schoolchildren; hence the sample size was not powered on any specific exposure or outcome. We used convenient sampling of SONA participants that were enrolled between March and August 2016, when the mental health sub-study was conducted. We aimed to recruit as many participants as possible from those enrolled into SONA therefore sampling was entirely based on convenience.

***Data analysis.*** Statistical analyses were conducted using STATA version 15 (StataCorp, College Station, Texas, USA). Participants’ characteristics were described using means and standard deviations for continuous variables, and proportions for categorical variables. Raw scores on neurocognitive tests were first described using means and standard deviations before the data were categorised into a binary variable. We compared neurocognitive scores based on each of the sociodemographic variables using group means. For each neurocognitive domain and for each age group, raw scores were converted into z-scores by dividing individual scores by the standard deviations in the respective domain. Neurocognitive impairment was defined as having a z-score of less than -2 in any of the domains, or a z-score of -1 in at least two domains. For the psychiatric disorders, binary diagnosis for each disorder was derived using a symptom count and other criteria as given by the CASI-5 and YI-4R scoring instructions.

Associations between neurocognitive impairment, with sociodemographic variables and psychiatric disorders were examined using crude and adjusted logistic regression (adjusting for each variable). Similarly, associations between psychiatric disorder and sociodemographic exposures were examined using crude and adjusted logistic regression analysis to generate odds ratios. For all analyses, the 95% confidence interval was determined.

## Results

### Participant sociodemographic characteristics

Of the 515 SONA participants seen, 322 participants (130 boys, 40.3%) ^27^ were enrolled and assessed for neurocognitive and psychiatric disorders, including children aged 5–11 years (n=158; 40.4%) and adolescents aged 12 to 17 years (n=164, 50.8%) ( Table 1). The sociodemographic characteristics are summarised in Table 1. 193(37.5%) SONA participants were excluded because they did not meet the eligibility criteria.

**Table 1.** Sociodemographic characteristics of participants (N=322).

Factor	Level	N (%)
Age (categorised)	Child (5-11 years) Adolescent(12-17yrs)	158(48.9) 164(50.8)
Sex	Boys Girls	130(40.3) 193(59.7)
Type of school funding	Government funded Privately owned	144(44.9) 177(55.1)
Economic status of school	Low High	158(49.2) 163(50.8)
*Grade	P1 – P4 P5 – P7 S1 – S4 S5 – S6	121(37.5) 69(21.4) 113(35) 6(1.9)
Father’s education	None Primary Secondary Tertiary	9(2.8) 92(28.7) 126(39.4) 93(29.1)
Mother’s education	None Primary Secondary Tertiary	3(1.0) 124(38.9) 118(36.9) 74(23.2)
Asthma status	Yes No	61(19.1) 258(80.9)

*Grades P1-P4 correspond with 6–9 years of age; P5-P7 with age 10–12years; S1-S with age 13–16 years; S5-S6 with 17–18 years.

### Description of neurocognitive abilities among the participants

A total of 321 participants had complete data on the Simultaneous Processing scale, the group mean score was 13.2 (s.d, 5.9), range 2–41. All 322 participants completed the Sequential Processing scale, their mean score was 14.4 (s.d, 4.4), range 3–26. Planning scale was completed by only 130 children, since we did not assess the adolescents on this scale. Mean score on this scale was 5.3 (s.d, 2.9) and range 1–13. Medians and interquartile ranges of these scores were also explored. These results are summarised in Table 2. Performance data on each of the scales had a nearly normal distribution (Supplementary Figure 1, *Extended data* ^27^)

**Table 2.** Descriptive summaries for performance on the neurocognitive measures.

Neurocognitive domain	N =322	Mean(SD)	Median (Interquartile range)	(Min, max)	skewness	kurtosis
Simultaneous processing scale	321	13.2(5.9)	12(4,31)	(2,41)	1.27	5.70
Sequential processing scale	322	14.2(4.4)	14(6,24)	(3,26)	0.29	2.48
Planning	130	5.3(2.9)	5(3,7)	(1,13)	0.67	2.91

Mean neurocognitive scores were compared based on sex, age, school type, school status, school grade and found differences between the groups. Boys performed better than girls in Simultaneous Processing (mean diff=2.5, p<0.001); and Sequential Processing (mean diff=1.3, p<0.001), but in Planning they (boys) had a lower mean score than girls (mean diff=-0.8, p<0.001).

Participants attending privately owned schools had higher scores than those in government-supported schools, and the differences were significant for all the domains: Simultaneous Processing (mean diff=1.5; p<0.001); Sequential Processing domains (mean diff=1.2; p<0.001), and Planning (mean diff=1.1; p<0.001) ( Table 3). Similarly, higher scores were observed in participants attending high economic status schools than those in lower status schools, and the differences were significant for Simultaneous Processing (mean diff=3.0; p<0.001); Sequential Processing domains (mean diff=2.4; p<0.001), and Planning (mean diff=1.5; p<0.001) ( Table 3).

**Table 3.** Comparison of neurocognitive scores based on sociodemographic variables.

		Simultaneous Processing scale	p-value	Sequential Processing scale	p-value	*Planning	p-value
**Sex**	Male Female	14.7 12.2	<0.001	14.6 13.9	<0.001	5.0 5.8	<0.001
**Age category**	Children Adolescents	14.4 12.1	<0.001	15.5 12.9	<0.001		
**Type of school**	Private Government	13.9 12.4	<0.001	14.7 13.5	<0.001	5.9 4.8	<0.001
**Economic status of school**	Low status High status	11.7 14.7	<0.001	12.9 15.3	<0.001	4.5 6.0	<0.001
**Grade**	P1 – P4 P5 – P7 S1 – S4 S5 – S6	14.3 13.3 12.0 12.1	<0.001	15.3 14.2 12.8 13.1	<0.001	5.4 5.4 - -	0.591
**Father’s education**	None Primary Secondary Tertiary	14.5 12.8 12.8 14.1	<0.001	15.1 14.0 13.8 14.8	<0.001	3 5.1 4.6 6.7	0.006
**Mother’s education**	None Primary Secondary Tertiary	12.8 13.1 12.8 14.1	<0.001	14.3 14.2 13.8 14.7	0.006	5.6 5.4 5.4 5.6	0.518
**Asthma status**	Yes No	12.9 13.3	0.281	13.8 14.3	0.102	5.2 5.5	0.142

*Planning was done only among children

Unexpectedly, children had higher scores than adolescents on Simultaneous Processing (mean diff=2.3, p<0.001) and Sequential Processing (mean diff=2.6, p <0.001) ( Table 3). In the same way, participants in lower classes (academic level) tended to have higher scores than those in higher classes and this was significant for Simultaneous Processing (p<0.001), and Sequential Processing (p<0.001). Children whose parents (father or mother) had tertiary education tended to have the highest scores. The differences in means were significant for Simultaneous Processing, Sequential Processing and Planning ( Table 3). We purposed to assess both the children and adolescents on all the subscales, however, the planning scale was erroneously missed for the adolescents.

### Prevalence of neurocognitive impairment among participants

**Figure 2.**
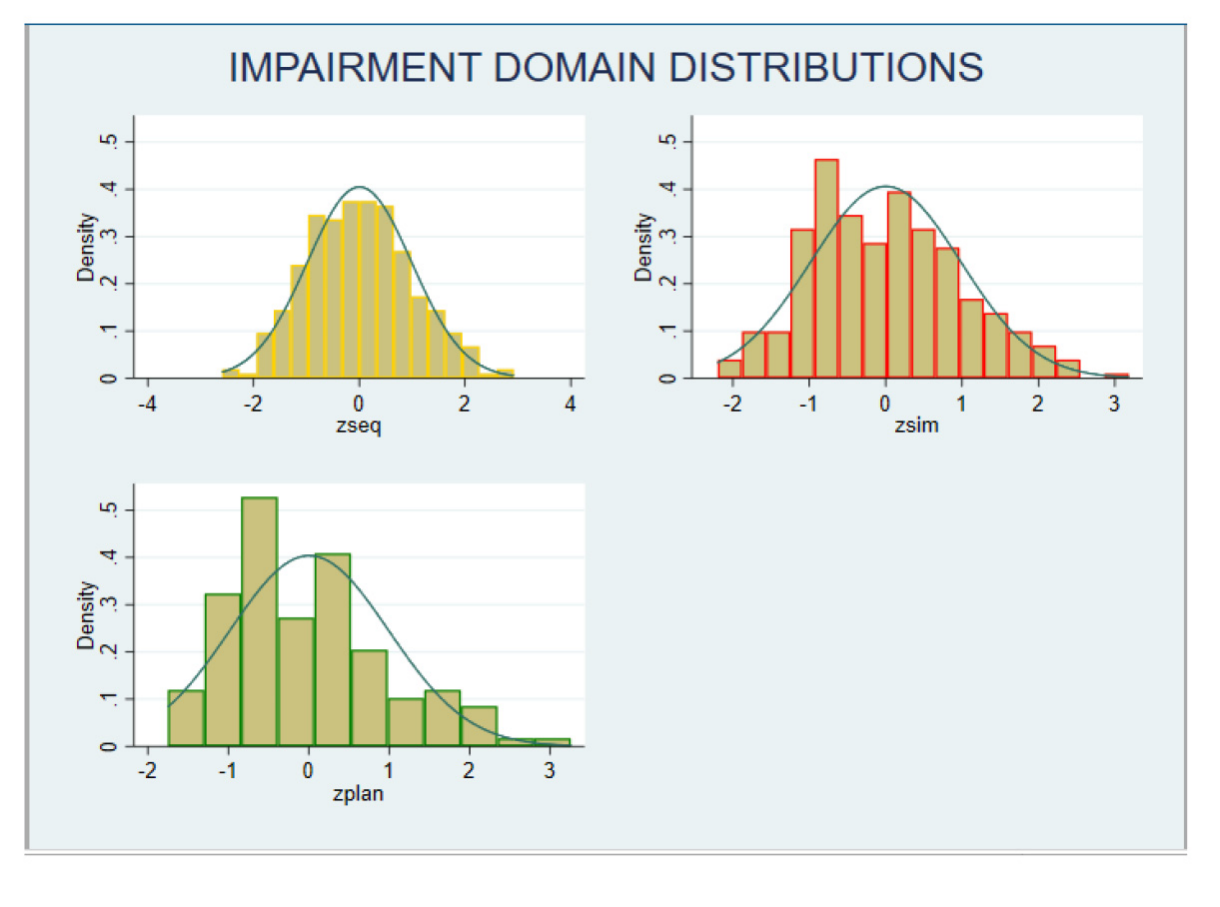
Distribution of neurocognitive impairment (z-scores). Zseq – Sequential Processing scale; Zsim – Simultaneous Processing scale; Zplan – Planning scale.

The z-scores on each neurocognitive domain showed a normal distribution ( Figure 2). Categorising performance data based on z-scores showed that, six participants had z-score of less than -2 in any domain, and 19 participants had z-score -1 in two or more domains. Hence, 25 participants (8%); 95% CI (5.5% -11.6%) were categorised as having neurocognitive impairment.

### Associations between sociodemographic factors and neurocognitive impairment

Academic level (grade) of the participant had a borderline significant association with neurocognitive impairment [adjusted odds ratio (aOR)=0.18; confidence interval (CI)= (0.03; 0.89); P=0.047]; the rest of the exposure variables were not significantly associated with neurocognitive impairment (p>0.05) ( Table 4).

**Table 4.** Factors associated with neurocognitive impairment among school children.

Factor	level	Crude OR (95%CI)	*Adjusted OR (95%CI)
***Sociodemographic characteristics***			
**Age (categorised)**	Child (5-11 years) Adolescent(12-17yrs)	1 0.58(0.25; 1.31) p=0.189	1 0.51(0.22; 1.19) p=0.120
**Sex**	Boys Girls	1 1.57(0.66; 3.72) p=0.307	1 1.81(0.75; 4.39) p=0.187
**Type of school funding**	Government Private	1 0.80(0.36; 1.78) p=0.583	1 0.74(0.33; 1.66) p=0.466
**Economic status of school**	Low High	1 0.58(0.25; 1.32) p=0.194	1 0.48(0.20; 1.13) P=0.156
**Grade**	P1 – P4 P5 – P7 S1 – S4 S5 – S6	1 0.32(0.09; 1.15) 0.40(0.15; 1.06) 1 p=0.061	1 0.18(0.03; 0.89) 0.14(0.03; 0.75) 1 p=0.047
**Mother’s highest education level ** **attained**	None Primary Secondary Tertiary	1 9.9(1.28; 76.6) 5.2(0.64; 42.7) 1 P=0.056	1 7.5(0.95; 59.6) 4.7(0.56; 40.35) 1 P=0.133
**Father’s highest education level** ** attained**	None Primary Secondary Tertiary	1 0.37(0.07; 2.11) 0.27(0.05; 1.49) 0.20(0.03; 1.23) P=0.318	1 0.22(0.03; 1.43) 0.20(0.03; 1.29) 0.13(0.02; 0.91) P=0.237
***Psychiatric illness factors***			
**Major depressive disorder**	Yes	0.62(0.08; 4.84) p=0.648	0.58(0.07; 4.58) P=0.606
**Attention deficit hyperactive disorder**	Yes	1.45(0.17; 12.02) P=0.733	1.15(0.13; 9.96) P=0.897
**Generalised anxiety disorder**	Yes	1.94(0.22; 16.74) P=0.547	1.44(0.16; 12.98) P=0.741
**At least one psychiatric disorder**	Yes	0.80(0.18; 3.57) P=0.770	0.92(0.19; 4.26) P=0.913
***Substance use***			
**Use of at least one substance**	Yes	2.94(0.56; 15.41) P=0.203	2.93(0.54; 15.69) P=0.210

OR=odds ratio, CI=confidence interval, P=primary, S=secondary; *association with each sociodemographic variable was adjusted for all the other sociodemographic variables in this table

### Prevalence of and risk factors for psychiatric disorders

Four psychiatric disorders MDD, ADHD, GAD, and SUD (tobacco, alcohol, marijuana) were found in this study population; the other disorders were not present. The prevalence of the four disorders was as follows: MDD was 5.9% (n=19); ADHD 2.8% (n=9); GAD 2.2% (n=7); and SUD 8.6% (n=14). Of the nine participants found to have ADHD, seven presented with the inattentive type while two had hyperactive-impulsive type. Prevalence of any psychiatric disorder was 9.3% (n=30), and was more common among children (12.0%) than adolescents (6.7%). There were no significant differences in the prevalence of psychiatric disorders between boys (8.5%) and girls (9.8%) (p=0.675).

We conducted crude and adjusted logistic regressions between having any psychiatric disorder and the sociodemographic variables. Asthma was found to be associated with MDD (AOR, 95%CI 2.71 1.02; 7.20) but not with the other disorders. None of the sociodemographic characteristics were significantly associated with having any psychiatric disorder (p>0.05) both in the crude and adjusted logistic regressions ( Table 5).

**Table 5.** Associations between psychiatric disorders and socio demographic characteristics ( *Adjusted OR; 95%CI).

		Major depressive disorder Adjusted OR; 95%CI *	Attention deficit hyperactive disorder Adjusted OR; 95%CI *	Generalized anxiety disorder Adjusted OR; 95%CI *	Substance abuse disorder	At least one psychiatric disorder (MDD,ADHD,GAD) Adjusted OR; 95%CIv
**Age** **(categorised)**	Child Adolescent	1 0.74(0.12;4.39) P=0.740	-	-	-	1 0.37(0.08;1.79) P=0.218
**Sex**	Male Female	1 2.10(0.64;6.83) P=0.215	1 0.62(0.14;2.72) P=0.533	1 1.42(0.30;6.58) P=0.655	1 1.01(0.29; 3.49) P=0.984	1 1.18(0.52;2.71) P=0.687
**Type of school** **funding**	Government Private	1 0.34(0.11;1.02) P=0.049	1 0.73(0.17;3.08) P=0.673	1 4.99(0.58;42.71) P=0.142	1 1.61(0.50; 5.20) P=0.422	1 0.71(0.32;1.59) P=0.411
**Economic** **status of** **school**	Low High	1 0.37(0.12;1.13) P=0.083	1 0.36(0.08;1.56) P=0.172	1 4.13(0.48;35.33) P=0.195	1 0.63(0.19; 2.27) P=0.511	1 0.59(0.26;1.34) P=0.210
**Grade**	P1 – P4 P5 – P7 S1 – S4 S5 – S6	1 1.57(0.33;7.38) 1.49(0.19;11.84) 12.60(0.96;164.8) P=0.1443	1 0.49(0.06;4.14) - - P=0.512	1 1.40(0.26;7.59) - - P=0.655	1 1 0.66(0.07; 6.17) 1 P=0.719	1 1.06(0.35;3.25) 1.05(0.17;6.35) 9.34(0.88;98.55) P=0.145
**Mother’s** **highest** **education** **level attained**	None Primary Secondary Tertiary	1 1.38(0.31;6.05) 1.62(0.39;6.72) - P=0.799	1 0.37(0.06;2.40) 1.12(0.21;6.07) - P=0.458	1 0.53(0.07;4.02) 1.60(0.25;10.39) - P=0.495	1 2.42(0.26; 22.76) 2.74(0.31; 23.69) 1 P=0.401	1 0.67(0.24;1.89) 1.01(0.37;2.78) - P=0.655
**Father’s** **highest** **education** **level attained**	None Primary Secondary Tertiary	1 0.46(0.04;4.72) 0.34(0.03;3.50) 0.21(0.02;2.50) P=0.582	1 0.39(0.03;4.53) 0.30(0.02;3.92) 0.29(0.02;3.82) P=0.794	1 0.80(0.11;6.05) 1.45(0.23;9.20) - P=0.810	1 1.28(0.30; 5.47) 0.56(0.14; 2.29) 1 P=0.541	1 1.14(0.12;10.35) 0.89(0.10;8.23) 0.80(0.08;7.53) P=0.919
**Asthma**	Non-asthmatic Asthmatic	1 2.71 (1.02; 7.20) P= 0.046	1 1.24(0.25;6.11) P=0.795	1 0.71(0.08; 6.03) P=0.755	1 -	1 2.01(1.05;3.11) P<0.001

OR=odds ratio, CI=confidence interval. P=primary. *Adjusted for other sociodemographic variables.

## Discussion

The main aim of the current study was to measure the prevalence of neurocognitive and psychiatric disorders in schoolchildren in Uganda using a sample of children drawn from the general population. The prevalence of neurocognitive impairment and any psychiatric disorder was 8% and 9.3%, respectively. Among the many psychiatric disorders examined in the sample, four were found to be prevalent, these were ADHD (all forms), GAD, MDD, and SUD (tobacco, marijuana, or illegal drugs). The burden of specific disorders varied, with substance use disorder presenting the highest burden at 8.6% followed by MDD (5.9%), ADHD (2.8%) and lowest for general anxiety (2.2%). The overall prevalence of psychiatric disorders in this sample of schoolchildren is less than the 20% reported globally, and as would be expected, less than the rates reported in children affected by HIV and war, however, it represents a significant burden of mental disorder in a general population that is assumed to be healthy. These results indicate that ideally in this population children and adolescents would benefit from routine screening for neurocognitive and psychiatric disorders, and provision of treatment for those found to be affected in line with the existing policy on routine screening. The government, through the Ministry of Health, could ensure that this policy is implemented. That said, routine screening for mental disorders in the entire (general) child and adolescent population would be expensive and maybe not feasible given the limited funds within which the Ministry of Health operates. It would perhaps be more practical and possibly more cost-effective to conduct targeted screening for psychiatric and neurocognitive disorders among those at risk and those showing signs of dysfunction in the identified mental health areas such as poor academic performance, social isolation, depressed mood, fear and anxiety, and conduct behaviour.

We explored associations between neurocognitive impairment, psychiatric disorders and sociodemographic and health factors to identify possible risk factors. Mean differences in neurocognitive scores (as a continuous variable) based on the different characteristics were observed and all were in the expected direction except the differences between children and adolescents, and between lower and higher academic class which were in the opposite direction. This finding was unexpected since developmentally adolescents should exhibit more mature cognitive skills including planning and inhibitory control than younger children. The surprising finding could be due to the tendency of some adolescents to take on deviant behaviour as they go through the self-identification that characterises the adolescent stage ^28^ and hence appearing to be more impulsive than young children.

We noted gender differences in the cognitive scores particularly where males performed better than females on sequential and simultaneous processing scales, while in planning, females performed better than the males. Gender differences in cognitive abilities have been widely studied using various tests, and have revealed differences in performance between males and females (girls and boys) with many showing a consistent pattern where females outscore males on the planning ability ^29, 30^. It is possible that females naturally have an advantage in planning over the males, therefore not surprising that these differences were observed in this study population.

However, adjusted logistic regressions analysis showed that among the factors examined, only having history of asthma was significantly associated with the mental disorders, and this was only with MDD. This might be because of the small number of participants with neurocognitive impairment and psychiatric disorder in the sample. Hence we are not able to identify other factors that underlie the burden of mental health disorders in this sample of children. We recommend larger studies to explore this topic further.

We acknowledge the following limitations. First, the study was conducted within a larger study and was limited to the few sociodemographic items that were assessed in the main study. In as much as the risk factors included in the analysis were based on previous literature, the availability of information on those variables in the SONA study also determined what exposure variable was included in the analyses. Apart from asthma which was the main exposure in the SONA study, other risk factors for the neurocognitive and psychiatric outcomes used i.e. age, sex, grade of schooling, type of school and maternal and father’s education and family socio-economic status were included both based on the theoretical grounds but also because data on these had been collected within the main study. Hence, as such, mother’s and father’s highest education level attained, the school type and school status (determined based on amount of school fees) that the child was attending were used as a proxy for socioeconomic status (SES). A more exhaustive measure of SES might have provided better discrimination with regards to the risk for neurocognitive impairment and psychiatric disorder and probably shown associations that have been reported in previous studies ^31^. Secondly, household exposures such as domestic violence, single parenthood and other family characteristics have been reported to be associated with mental health problems in children ^31– 35^; however, as explained above, there was limited collateral information regarding the family environment hence it was not possible to examine the role of household characteristics in this study. Thirdly, the modest sample size and cross-sectional design of the study further limited the capacity of the study to effectively examine risk factors associated with neurocognitive and psychiatric disorders. Of note, it was surprising that ADHD was not associated with neurocognitive impairment; this could have been due to the few cases of ADHD (n=9) and of neurocognitive impairment (n=25) that were found in the study population. Out of the 1702 participants who took part in SONA, 322 (18.9%) were included in the neurocognitive study, by convenient sampling. Ideally, a predetermined and randomly selected sample size would have provided a more representative sample. Although the decision to undertake the neurocognitive study was made from the beginning of the SONA study, actual data collection began much later (due to logistical reasons), therefore it was not possible to apply a systematic sample size calculation and random sampling. We opted for convenient sampling through which 37.5% of the SONA participants who were seen during the period were not included in neurocognitive assessments because their parents did not send back the consents or other exclusion criteria. There was therefore a risk for a selection and response bias since individuals who need help or who perceive themselves to have a health problem tend to volunteer to participate in studies of this nature. On the other hand, their interest in participating was probably out of a general curiosity to know about their children’s mental health status since such opportunities are not common in this setting. Lastly, the SONA study in which our study was nested was conducted in schools and within a peri-urban setting and hence there was no opportunity to examine the neurocognitive and psychiatric disorders in children and adolescents out of school and from rural settings. This may limit the generalisability of our findings to the general population.

Nonetheless, this study provided important data, and an epidemiological picture on the prevalence neurocognitive and psychiatric disorders of these conditions among children and adolescents in the general population in Uganda, and filled an important gap in the literature, particularly for tropical Africa. Future studies that recruit a much larger and random sample of participants are recommended.

Chronic diseases including HIV have been associated with poor neurodevelopmental outcomes in children ^19, 20, 36^. As part of the SONA study, all participants in this sub-study were tested for HIV and all were negative. The prevalence of asymptomatic malaria (thick smear) and worm infection was very low ^23^ and all children reported to be in good health (no complaints) at the time of assessments. A fifth of the participants reported history of asthma, and even though they were clinically in good health at the time of neurocognitive and psychiatric assessments, the data showed significant association between being asthmatic and major depressive disorder. Therefore, apart from those that reported history of asthma, the rest of this sample of children and adolescents were considered to be in good health status and hence would represent a general population.

## Conclusion

This study provides epidemiological data on the prevalence of neurocognitive and psychiatric disorders in the general population of children and adolescents in Uganda. The high prevalence of neurocognitive and mental disorders calls for investigation of risk factors using an epidemiological study, and for operationalisation of the child and adolescent mental health policy in Uganda through targeted screening of children and adolescents at risk ^1^. These data also provide very useful reference figures from the general population to compare with clinical populations including HIV, and to track trends in the burden of mental health problems over time. Having accurate data on the true burden of disease is vital for necessary interventions to be instituted in order to promote the mental wellbeing of children and adolescents. Larger epidemiological studies should be undertaken to generate more evidence on the burden and risk factors for mental health problems in children and adolescents in the general population, including exploring the role of the family and community environment.

## Data Availability

LSHTM Data Compass: CHAKA-SONA Normative dataset, https://doi.org/10.17037/DATA.00002434 ^27^. This project contains the following underlying data: Normative_dataset.txt (sociodemographic information, health and neurocognitive and psychiatric outcomes) Due to ethical considerations surrounding the sensitivity of the data in a vulnerable population, study consents limited the access to underlying data from this study. However, controlled access to the data posted in the above repository is permitted, subject to approval from the Uganda Virus Research Institute Research (UVRI) Ethics Committee and the Uganda National Council for Science and Technology. If access is approved, the applicant / their host institution will be asked to sign a Data Transfer Agreement, which includes conditions for the secure storage of data. Dataset use for further research will require additional ethics approval by the ethics committees that approved the original research. Access can be requested through the ‘Request access’ button in the above data project. The codebook (Normative_dataset_codebook.html) is available under the terms of the Creative Commons Attribution 4.0 International license (CC-BY 4.0). Data Compass: CHAKA-SONA Normative dataset, https://doi.org/10.17037/DATA.00002434 ^27^. This project contains the following extended data: CHAKA-SONA_support_documents.zip (questionnaires and participant consent forms) Data are available under the terms of the Data Sharing Agreement, as above. Supplementary_Figure1.pdf (Distribution of performance on neurocognitive scales – raw scores) Supplementary Figure 1 is available under the terms of the Creative Commons Attribution 4.0 International license (CC-BY 4.0).

## References

[1] MoH: Child and adolescent mental health policy guidelines. MoH, Kampala Uganda.2017. Reference Source

[2] Thumann BF Nur U Naker D : Primary school students’ mental health in Uganda and its association with school violence, connectedness, and school characteristics: A cross-sectional study. *BMC Public Health.* 2016;16:662. 10.1186/s12889-016-3351-z 27473040 PMC4966714

[3] Kessler RC Aguilar-Gaxiola S Alonso J : The global burden of mental disorders: An update from the WHO World Mental Health (WMH) surveys. *Epidemiol Psichiatr Soc.* 2009;18(1):23–33. 10.1017/s1121189x00001421 19378696 PMC3039289

[4] Kessler RC Aguilar-Gaxiola S Alonso J : The Global Burden of Mental Disorders: An Update from the WHO. *Epidemiol Psichiatr Soc.* 2011. 10.1017/s1121189x00001421 PMC3039289 19378696

[5] Okello J Onen TS Musisi S : Psychiatric disorders among war-abducted and non-abducted adolescents in Gulu district, Uganda: A comparative study. *Afr J Psychiatry (Johannesbg).* 2007;10(4):225–31. 10.4314/ajpsy.v10i4.30260 19588031

[6] Abbo C Kinyanda E Kizza RB : Prevalence, comorbidity and predictors of anxiety disorders in children and adolescents in rural north-eastern Uganda. *Child Adolesc Psychiatry Ment Health.* 2013;7(1):21. 10.1186/1753-2000-7-21 23841918 PMC3710504

[7] Kinyanda E Waswa L Baisley K : Prevalence of severe mental distress and its correlates in a population-based study in rural south-west Uganda. *BMC Psychiatry.* 2011;11:97. 10.1186/1471-244X-11-97 21651809 PMC3118177

[8] Nalugya-Sserunjogi J Rukundo GZ Ovuga E : Prevalence and factors associated with depression symptoms among school-going adolescents in Central Uganda. *Child Adolesc Psychiatry Ment Health.* 2016;10:39. 10.1186/s13034-016-0133-4 27800012 PMC5081935

[9] Kinyanda E Salisbury TT Levin J : Rates, types and co-occurrence of emotional and behavioural disorders among perinatally HIV-infected youth in Uganda: the CHAKA study. *Soc Psychiatry Psychiatr Epidemiol.* 2019;54(4):415–25. 10.1007/s00127-019-01675-0 30788554

[10] Kinyanda E Kizza R Abbo C : Prevalence and risk factors of depression in childhood and adolescence as seen in 4 districts of north-eastern Uganda. *BMC Int Health Hum Rights.* 2013;13:19. 10.1186/1472-698X-13-19 23561039 PMC3626891

[11] Mpango RS Rukundo GZ Muyingo SK : Prevalence, correlates for early neurological disorders and association with functioning among children and adolescents with HIV/AIDS in Uganda. *BMC Psychiatry.* 2019;19(1):34. 10.1186/s12888-019-2023-9 30665382 PMC6341558

[12] Biederman J : Attention-deficit/hyperactivity disorder: A selective overview. *Biol Psychiatry.* 2005;57(11):1215–20. 10.1016/j.biopsych.2004.10.020 15949990

[13] Faraone SV Perlis RH Doyle AE : Molecular genetics of attention-deficit/hyperactivity disorder. *Biol Psychiatry.* 2005;57(11):1313–23. 10.1016/j.biopsych.2004.11.024 15950004

[14] Martin J O’Donovan MC Thapar A : The relative contribution of common and rare genetic variants to ADHD. *Transl Psychiatry.* 2015;5(2):e506. 10.1038/tp.2015.5 25668434 PMC4445754

[15] Salvatore JE Aliev F Bucholz K : Polygenic Risk for Externalizing Disorders: Gene-by-Development and Gene-by-Environment Effects in Adolescents and Young Adults. *Clin Psychol Sci.* 2015;3(2):189–201. 10.1177/2167702614534211 25821660 PMC4371857

[16] Shrivastava A Desousa A : Resilience: A psychobiological construct for psychiatric disorders. *Indian J Psychiatry.* 2016;58(1):38–43. 10.4103/0019-5545.174365 26985103 PMC4776579

[17] Behere AP Basnet P Campbell P : Effects of family structure on mental health of children: A preliminary study. *Indian J Psychol Med.* 2017;39(4):457–463. 10.4103/0253-7176.211767 28852240 PMC5559994

[18] Currie K Bray I : Health inequalities, risky behaviours and protective factors in adolescents: an analysis of secondary survey data from the UK. *Public Health.* 2019;170:133–139. 10.1016/j.puhe.2019.03.001 31039467

[19] Vreeman RC Scanlon ML Marete I : Characteristics of HIV-infected adolescents enrolled in a disclosure intervention trial in western Kenya. *AIDS Care.* 2015;27 Suppl 1(sup1):6–17. 10.1080/09540121.2015.1026307 26616121 PMC4685612

[20] Vreeman RC Scanlon ML McHenry MS : The physical and psychological effects of HIV infection and its treatment on perinatally HIV-infected children. *J Int AIDS Soc.* 2015;18(Suppl 6):20258. 10.7448/IAS.18.7.20258 26639114 PMC4670835

[21] Mishra N Belbase M Shrestha D : Childhood neurological illness in Nepal. *J Nepal Health Res Counc.* 2010;8(1):55–62. 21879017

[22] Griggs D Stafford-Smith M Gaffney O : Policy: Sustainable development goals for people and planet. *Nature.* 2013;495(7441):305–7. 10.1038/495305a 23518546

[23] Mpairwe H Namutebi M Nkurunungi G : Risk factors for asthma among schoolchildren who participated in a case-control study in urban Uganda. *eLife.* 2019;8:e49496. 10.7554/eLife.49496 31729315 PMC6914334

[24] Mpango RS Kinyanda E Rukundo GZ : Prevalence and correlates for ADHD and relation with social and academic functioning among children and adolescents with HIV/AIDS in Uganda. *BMC Psychiatry.* 2017;17(1):336. 10.1186/s12888-017-1488-7 28938881 PMC5610431

[25] Gadow KD Sprafkin J Carlson GA : A *DSM-IV*-Referenced, Adolescent Self-Report Rating Scale. *J Am Acad Child Adolesc Psychiatry.* 2002;41(6):671–9. 10.1097/00004583-200206000-00006 12049441

[26] Bangirana P Seggane-Musisi Allebeck P : A preliminary examination of the construct validity of the KABC-II in Ugandan children with a history of cerebral malaria. *Afr Health Sci.* 2009;9(3):186–92. 20589149 PMC2887024

[27] Nampijja M Sembajjwe W Mpango RS : CHAKA-SONA Normative dataset. [Data Collection]. London School of Hygiene & Tropical Medicine, London, United Kingdom.2021. 10.17037/DATA.00002434

[28] Tsang SKM Hui EKP Law BCM : Positive identity as a positive youth development construct: A conceptual review. *ScientificWorldJournal.* 2012;2012:529691. 10.1100/2012/529691 22649296 PMC3353282

[29] Bardos AN Naglieri JA Prewett PN : Gender differences on planning, attention, simultaneous, and successive cognitive processing tasks. *J Sch Psychol.* 1992;30(3):293–305. 10.1016/0022-4405(92)90012-T 1556207

[30] Naglieri JA Rojahn J : Gender differences in planning, attention, simultaneous, and successive (PASS) cognitive processes and achievement. *J Educ Psychol.* 2001;93(2):430–437. 10.1037/0022-0663.93.2.430

[31] McLeod JD Shanahan MJ : Poverty, Parenting, and Children’s Mental Health. *Am Sociol Rev.* 1993;58(3):351–366. 10.2307/2095905

[32] Meltzer H Doos L Vostanis P : The mental health of children who witness domestic violence. *Child Fam Soc Work.* 2009;14(4):491–501. 10.1111/j.1365-2206.2009.00633.x

[33] Osofsky JD : Children Who Witness Domestic Violence: The Invisible Victims. *Soc Policy Rep.* 1995;9(3):1–20. 10.1002/j.2379-3988.1995.tb00035.x

[34] Hornor G : Domestic violence and children. *J Pediatr Health Care.* 2005;19(4):206–12. 10.1016/j.pedhc.2005.02.002 16010259

[35] Bramlett MD Blumberg SJ : Family structure and children’s physical and mental health. *Health Aff (Millwood).* 2007;26(2):549–58. 10.1377/hlthaff.26.2.549 17339685

[36] Mellins CA Malee KM : Understanding the mental health of youth living with perinatal HIV infection: Lessons learned and current challenges. *J Int AIDS Soc.* 2013;16(1):18593. 10.7448/IAS.16.1.18593 23782478 PMC3687078

