# Supplementary material for: Prevalence and correlates of neurocognitive impairment and psychiatric disorders among schoolchildren in Wakiso District, Uganda: a cross-sectional study
Source: Wellcome Open Res. 2022 Dec 7;6:217. Originally published 2021 Sep 2. [Version 3] doi: 10.12688/wellcomeopenres.17005.3 (PMC9975401; doi:10.12688/wellcomeopenres.17005.3)
Supplement: Peer review history

# Peer review: Reviewer response for version 3

**DOI:** 10.21956/wellcomeopenres.20630.r53647

**Published:** 2022-12-12

**Authors:** Joanna Martin

**Affiliations:** 1 MRC Centre for Neuropsychiatric Genetics and Genomics, Division of Psychological Medicine and Clinical Neurosciences, Cardiff University, Cardiff, UK

I have no further comments on this paper now.

Is the work clearly and accurately presented and does it cite the current literature?

Yes

If applicable, is the statistical analysis and its interpretation appropriate?

Yes

Are all the source data underlying the results available to ensure full reproducibility?

Yes

Is the study design appropriate and is the work technically sound?

Yes

Are the conclusions drawn adequately supported by the results?

Yes

Are sufficient details of methods and analysis provided to allow replication by others?

Partly

Reviewer Expertise:

Child psychiatry, neurodevelopment, mental health, epidemiology

I confirm that I have read this submission and believe that I have an appropriate level of expertise to confirm that it is of an acceptable scientific standard.

# Peer review: Reviewer response for version 2

**DOI:** 10.21956/wellcomeopenres.20360.r52556

**Published:** 2022-10-17

**Authors:** Rahul Shidhaye

**Affiliations:** 1 Department of Research, Pravara Institute of Medical Sciences, Loni, Maharashtra, India

Authors have satisfactorily addressed all my comments. I would like to thank the authors for same and congratulate them on undertaking and publishing this important work.

Is the work clearly and accurately presented and does it cite the current literature?

Yes

If applicable, is the statistical analysis and its interpretation appropriate?

Yes

Are all the source data underlying the results available to ensure full reproducibility?

Yes

Is the study design appropriate and is the work technically sound?

Yes

Are the conclusions drawn adequately supported by the results?

Yes

Are sufficient details of methods and analysis provided to allow replication by others?

Yes

Reviewer Expertise:

Global Mental Health, Implementation Research, Yoga, Epidemiology

I confirm that I have read this submission and believe that I have an appropriate level of expertise to confirm that it is of an acceptable scientific standard.

# Peer review: Reviewer response for version 2

**DOI:** 10.21956/wellcomeopenres.20360.r52555

**Published:** 2022-10-11

**Authors:** Joanna Martin

**Affiliations:** 1 MRC Centre for Neuropsychiatric Genetics and Genomics, Division of Psychological Medicine and Clinical Neurosciences, Cardiff University, Cardiff, UK

Thank you for responding to my previous comments. I am happy with the responses, except for the response to my comment #9, which I think has not been fully addressed yet. Although the authors noted that the only significant association between asthma and the studied disorders in this sample is MDD, the lack of significant associations with other disorders could simply be due to the small sample size, so my other suggestions could still be worthwhile to help provide additional information.

 Here is my previous comment: 

 9. The recruitment for this study was originally for a case-control study of asthma and therefore it seems the sample is over-represented for asthma (19.1% of the sample according to Table 1). There is some evidence that asthma is associated with ADHD and therefore this over-representation of asthma in the sample may have increased the prevalence of these other problems observed in the sample. This should be mentioned in the Discussion. The authors should also check what the prevalence results for the psychiatric disorders look like in the subsample without asthma.

Is the work clearly and accurately presented and does it cite the current literature?

Yes

If applicable, is the statistical analysis and its interpretation appropriate?

Yes

Are all the source data underlying the results available to ensure full reproducibility?

Yes

Is the study design appropriate and is the work technically sound?

Yes

Are the conclusions drawn adequately supported by the results?

Yes

Are sufficient details of methods and analysis provided to allow replication by others?

Partly

Reviewer Expertise:

Child psychiatry, neurodevelopment, mental health, epidemiology

I confirm that I have read this submission and believe that I have an appropriate level of expertise to confirm that it is of an acceptable scientific standard, however I have significant reservations, as outlined above.

# Response

**Published:** 2022-11-15

**Authors:** Margaret Nampijja

**Affiliations:** MRC/UVRI and LSHTM Uganda Research Unit, Uganda

We thank the reviewer for the comment raised about overrepresentation of asthma in the sample. We have checked prevalence of the psychiatric disorders among the asthmatic sub-sample and the non-asthmatic subsample and found a higher prevalence of major depressive disorder in the asthmatic group than the non-asthmatic group;  a higher rate of substance abuse in the non-asthmatic group but none in the asthmatic group. The prevalence of generalized anxiety disorders and ADHD were similar between the two groups.  Therefore asthma appears to drive the prevalence of MDD in the sample but not the other disorders.

 These results are now included in the revised manuscript in (Table 6) and in the discussion.

# Peer review: Reviewer response for version 1

**DOI:** 10.21956/wellcomeopenres.18777.r49168

**Published:** 2022-05-30

**Authors:** Rahul Shidhaye

**Affiliations:** 1 Department of Research, Pravara Institute of Medical Sciences, Loni, Maharashtra, India

This study aims to estimate the prevalence of neurocognitive impairment and psychiatric disorders in a sample schoolchildren and adolescents without HIV or other diagnosed health condition living in a peri-urban setting in Wakiso, Uganda. The study found that 8% participants had neurocognitive impairment, 5.9% had major depression, 2.8% had attention deficit hyperactivity disorder, 2.2% had generalized anxiety disorder, and 8.6% had substance use disorder.

1. In the first sentence of the abstract, it will be preferable to use the term, ‘general population’, or ‘community sample’ of schoolchildren instead of ‘healthy’ children.
2. The last sentence of the methods section of the abstract mentions, ‘prevalence and risk factors were determined using percentages and logistic regression’. It will be good to say ‘descriptive statistics’ instead of percentages and specify simple/multiple logistic regression.
3. In the results section of the abstract, please mention the risk factors which were not found to be associated with the outcomes.
4. The term, ‘unexpectedly high burden’ can be modified as we don’t know the expected burden of mental disorders and neurocognitive impairment in general population.
5. The study aimed to estimate prevalence of neurocognitive impairment and mental disorders in children in general population. If so, why only school children living in a peri-urban setting were selected? It is likely that these outcomes are worse in children from rural areas and not attending school. This limits the generalisability of the study findings and should be clearly mentioned in the limitations section.
6. The participants were selected using convenience sampling. This needs further clarification. Was there any sampling frame from which children were selected with some randomness in selection or was it entirely based on convenience?
7. What was the proportion of children excluded as parent was not available? This and the issue mentioned in previous comment are likely to introduce selection bias. This needs to be clearly mentioned in the limitations.
8. Please provide the participant flow chart in the beginning of the results section.
9. Is CASI-5 validated in the local language? Kindly provide the details.
10. Authors provide a list of disorders considered under the CASI-5 and YI-4-R. However, in the results section only four disorders are covered. Please explain.
11. What theoretical model/conceptual framework/DAG (Directed Acyclic Graph) guided the selection of risk factors for the outcomes studied? It is fine that only a few of the factors were included in the study and it will be good for the readers to know that the relation between the potential risk factors and the outcomes.
12. Why were adolescents excluded from the planning scale? This will be good for the readers to know.
13. It will be desirable to present median and inter-quantile range for descriptive summaries in table 2.
14. Figure 1 and 2 can be presented as supplementary material.

Is the work clearly and accurately presented and does it cite the current literature?

Yes

If applicable, is the statistical analysis and its interpretation appropriate?

Yes

Are all the source data underlying the results available to ensure full reproducibility?

Yes

Is the study design appropriate and is the work technically sound?

Yes

Are the conclusions drawn adequately supported by the results?

Yes

Are sufficient details of methods and analysis provided to allow replication by others?

Yes

Reviewer Expertise:

Global Mental Health, Implementation Research, Yoga, Epidemiology

I confirm that I have read this submission and believe that I have an appropriate level of expertise to confirm that it is of an acceptable scientific standard, however I have significant reservations, as outlined above.

# Response

**Published:** 2022-09-08

**Authors:** Margaret Nampijja

**Affiliations:** MRC/UVRI and LSHTM Uganda Research Unit, Uganda

**RESPONSES TO REVIEWER 2**

 This study aims to estimate the prevalence of neurocognitive impairment and psychiatric disorders in a sample schoolchildren and adolescents without HIV or other diagnosed health condition living in a peri-urban setting in Wakiso, Uganda. The study found that 8% participants had neurocognitive impairment, 5.9% had major depression, 2.8% had attention deficit hyperactivity disorder, 2.2% had generalized anxiety disorder, and 8.6% had substance use disorder.

 1. In the first sentence of the abstract, it will be preferable to use the term, ‘general population’, or ‘community sample’ of schoolchildren instead of ‘healthy’ children.

**RESPONSE:** Thank you for this observation, the word ‘general’ has been adopted replace health population in the abstract and in the main text.

 2. The last sentence of the methods section of the abstract mentions, ‘prevalence and risk factors were determined using percentages and logistic regression’. It will be good to say ‘descriptive statistics’ instead of percentages and specify simple/multiple logistic regression.

**RESPONSE:**  Thank you for the advice. We have made the necessary modification to the sentence. We used univariable and then multivariable logistic regression to examine the risk factors. This too has now been clarified in the methods section of the abstract. [Page 3]

 3. In the results section of the abstract, please mention the risk factors which were not found to be associated with the outcomes.

**RESPONSE:** These exposure variables including asthma, age, sex, grade of schooling, type of school and maternal and father’s education and family socio-economic status)  have now been inserted.[Page 3]

 4. The term, ‘unexpectedly high burden’ can be modified as we don’t know the expected burden of mental disorders and neurocognitive impairment in general population.

**RESPONSE:** The word “unexpectedly” has been replaced with “relatively” [Page 3]

 5.The study aimed to estimate prevalence of neurocognitive impairment and mental disorders in children in general population. If so, why only school children living in a peri-urban setting were selected? It is likely that these outcomes are worse in children from rural areas and not attending school. This limits the generalisability of the study findings and should be clearly mentioned in the limitations section.

**RESPONSE:** Thank you for highlighting this limitation. The SONA study in which our study was nested was conducted in schools and within a peri-urban setting and hence there was no opportunity to examine the neurocognitive and psychiatric disorders in children and adolescents out of school and from rural settings. This may limit the generalisability of our findings to the general population. This limitation has been acknowledged. [Page 16]

 6. The participants were selected using convenience sampling. This needs further clarification. Was there any sampling frame from which children were selected with some randomness in selection or was it entirely based on convenience?

**RESPONSE**: We used convenient sampling of SONA participants that were enrolled between March and August 2016, when the mental health sub-study was conducted, and aimed to recruit   as many participants as possible from those enrolled into SONA, therefore sampling was entirely by convenience. The ‘entirely by convenience’ has been highlighted under sample size section. [Page 7].

 7.What was the proportion of children excluded as parent was not available? This and the issue mentioned in previous comment are likely to introduce selection bias. This needs to be clearly mentioned in the limitations.

**RESPONSE**: SONA study sample size (during the time of the CHAKA Normative study =515 participants)

 The number of participants who met the entry criteria for the CHAKA normative study (322 participants). 

 Therefore, the number and proportion of children excluded (who never met entry criteria; parent was not available) was 193 (37.5%). We recognize that this could have created a selection bias and hence a bias in the findings reported. The number is clarified on page 7, and the limitation has been acknowledged in the limitation section [page 16].

 8. Please provide the participant flow chart in the beginning of the results section.

**RESPONSE**: A flow chart (Figure 1) showing how the participants were selected from the SONA study has been inserted in the methods section. We felt that the flow chart fits more appropriately within the description of participant selection.

 9. Is CASI-5 validated in the local language? Kindly provide the details.

**RESPONSE**: Yes, the CASI-5 was culturally adapted and translated in the local language(Luganda) that is predominantly used in the study setting. This has been indicated in the methods section. [Page 6]

 10. Authors provide a list of disorders considered under the CASI-5 and YI-4-R. However, in the results section only four disorders are covered. Please explain.

**RESPONSE**: We would like to clarify that the whole spectrum of disorders assessed by the CASI-5 and YI-4-R were examined in the study population. However only those that were present i.e. ADHD, MDD, GAD & SUD were included in the analysis. This has been clarified both in the methods and results section.[Page 6]

 11. What theoretical model/conceptual framework/DAG (Directed Acyclic Graph) guided the selection of risk factors for the outcomes studied? It is fine that only a few of the factors were included in the study and it will be good for the readers to know that the relation between the potential risk factors and the outcomes.

**RESPONSE:** The rational for the selection of risk factors that were included on the analysis was based on literature but also on the availability of information on those variables in the SONA study. In addition to asthma which was the main exposure in the main study, other risk factors including age, sex, grade of schooling, type of school and maternal and father’s education and family socio-economic status included based on scientific(theoretical) relevance and because the data on these were available in the main SONA study. Since the study conducted within a larger study, we were limited to the few sociodemographic variables that were assessed in the main study. This limitation has been expanded under the discussion section. [Page 15]

 12.Why were adolescents excluded from the planning scale? This will be good for the readers to know.

**RESPONSE: **We purposed to assess both the children and adolescents on all the subscales, however, the planning scale was erroneously missed for the adolescents. This has been indicated on page 10.

 13. It will be desirable to present median and inter-quantile range for descriptive summaries in table 2.

**RESPONSE: **We had chosen Mean(SD) because of the spread of the data. However, with your advice, we have also included the median(IQR) of the same. [Table 2]

                                                          

 Figure 1 and 2 can be presented as supplementary material.

**RESPONSE:** We have taken your advice and that of the first reviewer, and moved the two Figures (now 2 and 3) Figures to the data repository as extended data. This is the equivalent of supplementary material for the Wellcome Open Research Journal.

# Peer review: Reviewer response for version 1

**DOI:** 10.21956/wellcomeopenres.18777.r45753

**Published:** 2021-09-13

**Authors:** Joanna Martin

**Affiliations:** 1 MRC Centre for Neuropsychiatric Genetics and Genomics, Division of Psychological Medicine and Clinical Neurosciences, Cardiff University, Cardiff, UK

This is an epidemiological study examining the prevalence of neurocognitive impairments and psychiatric disorders in 322 school children aged 5-17 years old in Uganda. The results indicate that 8% of the sample had neurocognitive impairments and 9.3% had at least one psychiatric disorder. I have the following minor suggestions for improvement:

1. The authors describe the sample as “healthy” or “assumed healthy” in the abstract, introduction and discussion. This should be replaced with terms such as “general population” or “reference population” or “community sample” to be more accurate as we cannot assume that a sample of school children will be completely healthy. I would also suggest replacing “normal population” (in Discussion) with one of the terms above.
2. In the method, the authors note that the CASI-5 and YI-4-R can be used to assess many different psychiatric conditions but they focused only on ADHD, MDD, GAD & SUD. It later appears that this was because none of the other conditions were present in the sample. Can the authors clarify in the Method that they examined each of the conditions and only mention that they then focused on the specific conditions in the Results? The Discussion and abstract should also explicitly state that other disorders were also assessed but not detected in the sample.
3. Please state how the binary diagnoses were derived on the basis of the CASI-5 and YI-4-R. For example, how many items were used, was impairment or other clinical features (such as duration, onset) considered? How was information from multiple informants combined?
4. What age were the participants who completed the YI-4-R? Presumably very young children did not complete this self-reported measure.
5. Also please state briefly in the Results which types of SUD problems were present in the sample and how many children had which subtype of ADHD.
6. Table 5 does not include SUD- why is that? Surely SUD was associated with age, as younger children were not affected?
7. Can you please add a note to Table 1 indicating what ages the school grades correspond to for those unfamiliar with the categories P1-P4 etc.?
8. Figures 1 & 2: a few changes to these figures would be helpful: Figure 1 can be moved to the supplement or removed as it is of less interest than Figure 2 presenting the z-scores. It would also be helpful if the authors could change these figures to plot frequency not density on the y-axis.
9. The recruitment for this study was originally for a case-control study of asthma and therefore it seems the sample is over-represented for asthma (19.1% of the sample according to Table 1). There is some evidence that asthma is associated with ADHD (e.g. https://onlinelibrary.wiley.com/doi/full/10.1111/j.1398-9995.2011.02648.x) and other mental health problems (e.g. https://www.mdpi.com/2076-328X/9/7/78) and therefore this over-representation of asthma in the sample may have increased the prevalence of these other problems observed in the sample. This should be mentioned in the Discussion. The authors should also check what the prevalence results for the psychiatric disorders look like in the subsample without asthma.
10. As the authors point out, one limitation is the low statistical power in the analyses. This is also relevant to the analysis examining the association between neurocognitive impairment and diagnoses. It is surprising that for example ADHD is not associated with neurocognitive impairment but the sample is quite small (only 9 children with ADHD and only 25 had neurocognitive impairment). This limitation should be expanded on so that the lack of associations between the variables assessed is not over-interpreted by readers.

Is the work clearly and accurately presented and does it cite the current literature?

Yes

If applicable, is the statistical analysis and its interpretation appropriate?

Yes

Are all the source data underlying the results available to ensure full reproducibility?

Yes

Is the study design appropriate and is the work technically sound?

Yes

Are the conclusions drawn adequately supported by the results?

Yes

Are sufficient details of methods and analysis provided to allow replication by others?

Partly

Reviewer Expertise:

Child psychiatry, neurodevelopment, mental health, epidemiology

I confirm that I have read this submission and believe that I have an appropriate level of expertise to confirm that it is of an acceptable scientific standard, however I have significant reservations, as outlined above.

# Response

**Published:** 2022-09-08

**Authors:** Margaret Nampijja

**Affiliations:** MRC/UVRI and LSHTM Uganda Research Unit, Uganda

**RESPONSES TO REVIEWER 1**

 This is an epidemiological study examining the prevalence of neurocognitive impairments and psychiatric disorders in 322 school children aged 5-17 years old in Uganda. The results indicate that 8% of the sample had neurocognitive impairments and 9.3% had at least one psychiatric disorder. I have the following minor suggestions for improvement:

 1. The authors describe the sample as “healthy” or “assumed healthy” in the abstract, introduction and discussion. This should be replaced with terms such as “general population” or “reference population” or “community sample” to be more accurate as we cannot assume that a sample of school children will be completely healthy. I would also suggest replacing “normal population” (in Discussion) with one of the terms above.

**RESPONSE**: We thank the reviewer for this observation; we have adopted their suggestion and used “general” instead of “healthy” population, in the abstract  [Page 3]

  2. In the method, the authors note that the CASI-5 and YI-4-R can be used to assess many different psychiatric conditions but they focused only on ADHD, MDD, GAD & SUD. It later appears that this was because none of the other conditions were present in the sample. Can the authors clarify in the Method that they examined each of the conditions and only mention that they then focused on the specific conditions in the Results? The Discussion and abstract should also explicitly state that other disorders were also assessed but not detected in the sample.

**RESPONSE**: We would like to clarify that the whole spectrum of disorders assessed by the CASI-5 and YI-4-R were examined in the study population. However only those that were present i.e. ADHD, MDD, GAD & SUD were included in the analysis. This has been clarified both in the methods and results section. [Page 6] 

 3. Please state how the binary diagnoses were derived on the basis of the CASI-5 and YI-4-R. For example, how many items were used, was impairment or other clinical features (such as duration, onset) considered? How was information from multiple informants combined?

**RESPONSE: **The binary diagnosis for each disorder was derived using a symptom count and other criteria as given by the CASI-5 and YI-4R scoring instructions. [Page 7].

 4.What age were the participants who completed the YI-4-R? Presumably very young children did not complete this self-reported measure.

**RESPONSE:** The YI-4 R was self-administered to youths aged 12–18 years. Younger children did not complete this self-reported measure. This has been clarified in the methods in the second paragraph under ***Assessing for psychiatric disorders***. [Page 6]

 5. Also please state briefly in the Results which types of SUD problems were present in the sample and how many children had which subtype of ADHD.

**RESPONSE: **The substances looked at were tobacco, alcohol, marijuana. Of the nine participants found to have ADHD, seven presented with the inattentive type while two had hyperactive-impulsive type. This has been included in the revised manuscript [page 12].

 6.Table 5 does not include SUD- why is that? Surely SUD was associated with age, as younger children were not affected?

**RESPONSE:** Data on SUD has been added to Table 5.

 7.Can you please add a note to Table 1 indicating what ages the school grades correspond to for those unfamiliar with the categories P1-P4 etc.?

**RESPONSE:** Grades P1-P4 correspond with 6-9 years of age; P5-P7 with age 10-12years; S1-S4 with age 13-16 years; S5-S6 with 17-18 years. These have now been indicated below Table 1. [Page 8]

 8. Figures 1 & 2: a few changes to these figures would be helpful: Figure 1 can be moved to the supplement or removed as it is of less interest than Figure 2 presenting the z-scores. It would also be helpful if the authors could change these figures to plot frequency not density on the y-axis.

**RESPONSE:** We have taken your advice and that of the second reviewer, and moved Figures 1 and 2 to the data repository as extended data. This is the equivalent of supplementary material for the Wellcome Open Research Journal.

 The y-axis in the normal distribution represents the “probability density”, which intuitively shows the chance of obtaining values near the corresponding points on the x-axis.

 9. The recruitment for this study was originally for a case-control study of asthma and therefore it seems the sample is over-represented for asthma (19.1% of the sample according to Table 1). There is some evidence that asthma is associated with ADHD (e.g. https://onlinelibrary.wiley.com/doi/full/10.1111/j.1398-9995.2011.02648.x) and other mental health problems (e.g. https://www.mdpi.com/2076-328X/9/7/78) and therefore this over-representation of asthma in the sample may have increased the prevalence of these other problems observed in the sample. This should be mentioned in the Discussion. The authors should also check what the prevalence results for the psychiatric disorders look like in the subsample without asthma.

**RESPONSE:** We have examined the association between asthma and the psychiatric disorders. Asthma was found to be associated with MDD (AOR, 95%CI 2.71 1.02; 7.20) but not with the other disorders. [Page 13].

 10. As the authors point out, one limitation is the low statistical power in the analyses. This is also relevant to the analysis examining the association between neurocognitive impairment and diagnoses. It is surprising that for example ADHD is not associated with neurocognitive impairment but the sample is quite small (only 9 children with ADHD and only 25 had neurocognitive impairment). This limitation should be expanded on so that the lack of associations between the variables assessed is not over-interpreted by readers.

**RESPONSE:** We thank the reviewer for highlighting this important limitation, and as advised, we have expanded the explanation on this limitation. [Page 15]
